# In vivo imaging of metabolic heterogeneity across three endpoints relevant to aggressive breast cancer

**DOI:** 10.1093/pnasnexus/pgag027

**Published:** 2026-02-10

**Authors:** Victoria W D’Agostino, Michelle Kwan, Adelle Yong, Kira Grossman, Enakshi D Sunassee, Megan C Madonna, Matthew Hirschey, Gregory M Palmer, Nirmala Ramanujam

**Affiliations:** Department of Biomedical Engineering, Duke University, Durham, NC 27708, USA; Department of Biology, Duke University, Durham, NC 27708, USA; Department of Biology, Duke University, Durham, NC 27708, USA; Department of Biomedical Engineering, Vanderbilt University, Nashville, TN 37235, USA; Department of Biomedical Engineering, Duke University, Durham, NC 27708, USA; Department of Biomedical Engineering, Duke University, Durham, NC 27708, USA; Department of Medicine, Duke University, Durham, NC 27708, USA; Department of Radiation Oncology, Duke University, Durham, NC 27708, USA; Department of Biomedical Engineering, Duke University, Durham, NC 27708, USA; Department of Pharmacology and Cancer Biology, Duke University, Durham, NC 27708, USA

**Keywords:** tumor metabolism, breast cancer, fluorescence microscopy, fatty acid oxidation, glycolysis

## Abstract

Triple-negative breast cancer (TNBC) is an aggressive subtype of breast cancer with poor prognosis and a high likelihood of recurrence. Residual disease after therapy is a key predictor of recurrence, often driven by intratumoral metabolic heterogeneity. Accumulating evidence indicates that tumors are able to shift between glycolysis and oxidative metabolism and alter nutrient preferences to sustain growth and resist therapy. We have developed a in vivo microscope that enables near-simultaneous measurements of fluorescent metabolic surrogates of glucose, fatty acids, and oxidative phosphorylation through a combination of spectral separation and sequential delivery schemes. Widefield imaging with uniform illumination across the entire tumor landscape (5 mm × 5 mm) informs on the spatial distribution of these metabolic probes. We used this technology to investigate metabolic heterogeneity of a murine model of TNBC (4T1 tumor line) and normal mammary tissues that have distinctly different metabolic pathways. Mammary tissues relied primarily on oxidative metabolism and showed high levels of glucose and fatty acid uptake across the entire imaging area reflecting a single metabolic phenotype. Though tumors were predominantly glycolytic, they displayed a heterogeneous distribution of nutrient preferences with regions dominated by either fatty acid uptake, glucose uptake, or both. Taken together, this work highlights the importance of not only capturing multiple metabolic endpoints but also investigating their spatial relationships to understand heterogeneity in key substrates and metabolic pathways for energy production in vivo.

Significance Statement This study describes an imaging system that images metabolic heterogeneity in living tumors, enabling deeper insights into the metabolic underpinnings of treatment resistance in breast cancer. The imaging platform captures multiple metabolic endpoints, enables longitudinal imaging, and maps the heterogeneity in key substrates and metabolic pathways for energy production in vivo. This technology fills a persistent gap in preclinical imaging of tumor metabolism—identifying metabolic vulnerabilities to target and timing interventions to maximize treatment response.

## Introduction

Breast cancer is the second most diagnosed cancer worldwide and the second leading cause of cancer death in women, resulting in over 660,000 deaths worldwide annually (1). Prognosis is highly dependent on molecular subtype, with triple-negative breast cancer (TNBC) having the lowest 5-year survival rate of 8–16%, due in large part to treatment resistance and recurrence (2–6). Patients who experience a pathological complete response to treatment of primary tumors experience lower mortality rates than patients with residual disease after treatment (6, 7). Unfortunately, pathological complete response at the time of surgery following neoadjuvant treatment can be as low as 35% (6–8). The presence of residual disease remaining after preoperative systemic therapy is indicative of a 20–30% higher risk of recurrence (8, 9). Incomplete response to treatment, resulting in residual disease, is responsible for recurrence and metastasis (7, 9). Recurrent or metastatic disease is the most common cause of breast cancer death in patients (9–13).

The spatial organization of a tumor and heterogeneity across its microenvironment can drive treatment resistance via the survival of cellular subpopulations termed residual disease. Residual disease leads to recurrence and mortality (14–16). Single-cell profiling and whole-genome amplification have shown that population heterogeneity within a tumor may be caused by subpopulations of cells with genotypic and phenotypic differences that render them either treatment resistant or treatment sensitive. Genomic, epigenomic, transcriptomic, and proteomic changes can give rise to metabolic differences occurring in different parts of the tumor (17). Further differences in response to treatment may also arise from differential access to nutrients due to the spatial organization of tumor cells and blood vessels, creating nutrient-deplete or nutrient-replete regions within the tumor (18–21).

Work in this field has established that metabolic heterogeneity leads to flexibility in substrate usage, enabling tumors to resist treatment and recur (12–15). The oxidation of not only glucose but also fats and amino acids fuels oxidative phosphorylation (OXPHOS) (21–26). Alterations in lipid metabolism in cancers include increased uptake of lipids and increased fatty acid oxidation (FAO) (25, 26). Cancer-associated adipocytes can communicate with and modify cells in the tumor, which affects metabolic pathways (26). Breast cancer, situated in the adipocyte-replete breast environment, opportunistically uses fatty acid metabolism as a fuel source (23). Studies show increased survival in TNBC cells with increased FAO (13, 21). When a metabolic switch occurs, it can signal the probability of patient response to treatment (15).

Characterization of a metabolic switch between glycolysis and oxidative phosphorylation and the substrates they rely on requires a methodology capable of capturing multiple metabolic endpoints in vivo, a gap that exists between in vitro cellular assays and whole-organ imaging modalities like positron emission tomography (PET) and MR(S)I (16). PET imaging with tracers undoubtedly provides valuable clinical information for tumor staging and monitoring (16). However, the spatial resolution of PET is insufficient to capture tumor metabolic heterogeneity (16). Further, PET cannot image multiple endpoints simultaneously from the same tissue site (16). Optical imaging with fluorescent indicators is uniquely positioned to complement techniques such as PET (16). It can bridge the resolution gap, providing micron-level spatial resolution with millimeter-scale fields of view, and enable near-simultaneous measurement of multiple metabolic endpoints through a combination of spectral separation and sequential delivery schemes (16).

We have previously validated the use of three fluorophores for in vivo imaging of metabolism. The three fluorophores are TMRE (tetramethylrhodamine ethyl ester), 2-NBDG (2-[*N*-(7-nitrobenz-2-oxa-1,3-diazol-4-yl) amino]-2-deoxy-D-glucose), and Bodipy FL C16 (4,4-difluoro-5,7-dimethyl-4-bora-3a,4a-diaza-s-indacene-3-hexadecanoic acid) (27–33). TMRE is a cationic dye that accumulates in mitochondria proportional to mitochondrial membrane potential (29–31, 34). The glucose analog 2-NBDG, transported via GLUT proteins and phosphorylated by hexokinase, is a marker of glucose uptake (27). Bodipy FL C16 behaves similarly to unlabeled palmitate, the most common long-chain fatty acid in the human body, and it is taken up through fatty acid transport proteins on the cell surface (28). Bodipy FL C16 can report on fatty acid uptake because the fluorescent label on the 16th carbon of the palmitate cannot be metabolized (28, 35). All three have been robustly validated by our group through in vitro and in vivo studies (27–33) to verify that they report on biologically meaningful metabolic endpoints in vivo.

We have validated TMRE as a surrogate for oxidative phosphorylation through chemical perturbation studies using CCCP (a mitochondrial uncoupler), demonstrating the expected decrease in TMRE signal following membrane potential dissipation in both tumor and normal tissues (31). We have also demonstrated significant decreases in TMRE uptake under hypoxic conditions consistent with reduced oxidative phosphorylation in oxygen-depleted environments (31). Similarly, we have demonstrated that 2-NBDG is inversely related to the presence of glucose (owing to competition) (27) and increases in oxygen-depleted tissue environments (30, 31).

Our group has shown that Bodipy FL C16 fluorescence reflects biological fatty acid processing rather than nonspecific binding or passive accumulation (28). Specifically, we have demonstrated 2- to 4-fold increases in uptake of Bodipy FL C16 with the overexpression of the MYC oncogene and corresponding decreases upon MYC down-regulation that correlates with reduced fatty acid–binding protein 3 (FATP3) expression, which is a key protein in fatty acid transport and metabolism (28). This is consistent with independent stable isotope uptake assays (28). We also validated Bodipy FL C16 specificity through pharmacological inhibition with perphenazine, a Food and Drug Administration–approved fatty acid transport protein (FATP) inhibitor, demonstrating significant decreases in uptake both in vitro and in vivo (28). Additionally, prior literature demonstrates that when cells were treated with Bodipy FL C16–labeled fatty acids, subsequent lipid extraction and high performance liquid chromatography (HPLC)-based lipidomic separation revealed fluorescence signals within specific lipid classes, confirming incorporation of the probe into downstream lipid species (36).

The goal of the current study was to develop a co-delivery methodology to capture TMRE, 2-NBDG, and Bodipy FL C16 fluorescence on a pixel-by-pixel basis across the entire tissue landscape. The challenge with co-delivering all three fluorophores is that there is spectral overlap between the two substrate reporters, 2-NBDG and Bodipy FL C16. We show that by combining spectral unmixing with a microscope equipped with just four excitation, emission wavelength pairs, we are able to effectively separate the contribution of 2-NBDG and Bodipy FL C16 when they are simultaneously delivered. We have previously shown that TMRE can be co-delivered with either 2-NBDG (29–31) or Bodipy FL C16 without optical or biological crosstalk (32, 33). Therefore, we were able to readily incorporate TMRE into our platform to co-localize images of glucose uptake, fatty acids, and mitochondrial membrane potential. Imaging across the entire tumor microenvironment with this microscope provided important insights into heterogeneity of nutrients (glucose, fatty acids, or both) and metabolic pathways utilized for energy production.

## Results

### Optical crosstalk between overlapping excitation and emission spectra of Bodipy FL C16 and 2-NBDG can be mitigated using spectral unmixing

To enable co-delivery of all fluorophores, we previously developed a spectral unmixing approach that uses a 100-point fluorescence spectrum to separate the contribution of 2-NBDG and Bodipy FL C16 (33) in tissue phantoms. Our phantom studies (Table S1) also demonstrated that varying the optical properties over a wide range had negligible effects on the fluorescent probes (32, 33). To adapt this approach to imaging, we used the original 100-point spectrum to determine the minimum number of excitation–emission wavelength pairs required to separate the contributions of 2-NBDG and Bodipy FL C16 in a mixed phantom (Table S2). We concluded that unmixing a four-point spectrum (four excitation–emission wavelength pairs) could faithfully recapitulate the results achieved with spectrally unmixing the 100-point spectrum.

**Fig. 1.**
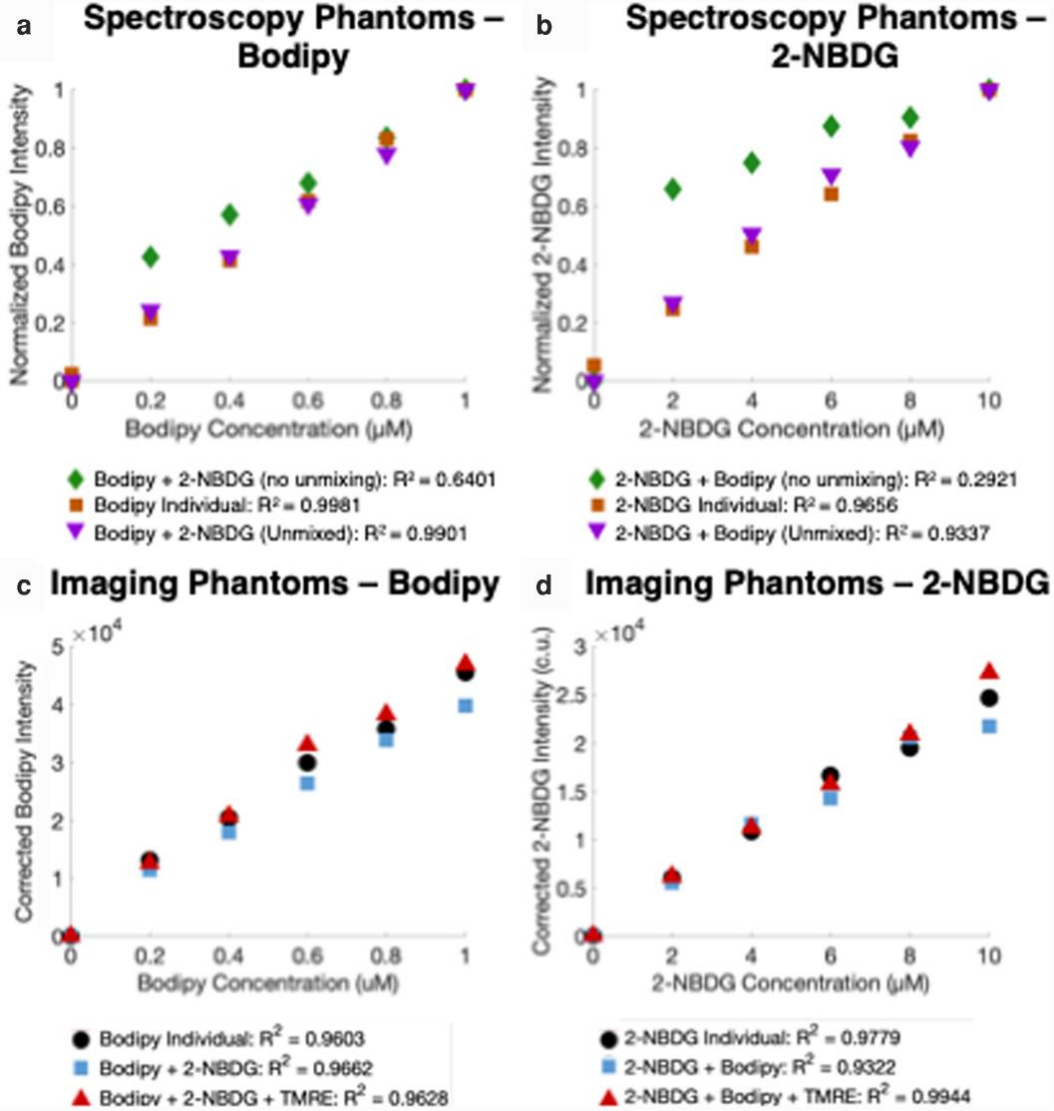
Optical crosstalk between overlapping excitation and emission spectra of Bodipy FL C16 and 2-NBDG can be mitigated via spectral unmixing. In (a) and (b), we selected the proposed wavelength ranges from the full fluorescence spectra (measured with spectroscopy) that most closely simulated the ranges we would capture with our imaging system. a) Bodipy FL C16 was prepared individually in a series of phantoms increasing in intensity linearly from 0.2 to 1 µM (squares). Individual Bodipy FL C16 (squares) was compared with Bodipy FL C16 mixed with a constant concentration of 2-NBDG both before unmixing (diamonds) and after unmixing (triangles). b) 2-NBDG was prepared individually in a series of phantoms increasing in intensity linearly from 2 to 10 µM (squares). Individual 2-NBDG (squares) was compared with 2-NBDG mixed with a constant concentration of Bodipy FL C16 both before unmixing (diamonds) and after unmixing (triangles). Next, we created homogenous phantoms and imaged them (c and d). c) Bodipy FL C16 was prepared individually in a series of phantoms increasing in intensity linearly from 0.2 to 1 µM (circles). Individual Bodipy FL C16 (circles) was compared with Bodipy FL C16 mixed with a constant concentration of 2-NBDG (squares) and Bodipy FL C16 mixed with a constant concentration of both 2-NBDG and TMRE (triangles) after unmixing was applied. d) 2-NBDG was prepared individually in a series of phantoms increasing in intensity linearly from 2 to 10 µM (circles). Individual 2-NBDG (circles) was compared with 2-NBDG mixed with a constant concentration of Bodipy FL C16 (squares) and 2-NBDG mixed with a constant concentration of both Bodipy FL C16 and TMRE (triangles) after unmixing was applied. All phantoms have a constant ${\;}_{s}^{\;}$ (mean ${\;}_{s}^{\;}$ of 10 cm^−1^ over a wavelength range from 500 to 600 nm) and no absorber. All data reported are a product of the coefficient matrix output from the unmixing algorithm and the appropriate reference value from the 470 nm LED 535 nm emission filter pair, excluding the data labeled “no unmixing,” which is raw intensity values. Data comparing mixed and unmixed data are normalized to the max value to show the linear relationship on a similar scale (a, b). Corrected units are used to denote unmixed intensity (c, d).

Figure 1 shows the validation results of the unmixing strategy, from the fluorescence spectroscopy data (Table S1) and widefield fluorescence images (Table S2), respectively of tissue phantoms. Before unmixing, the *R*^2^ value for Bodipy FL C16 (mixed with 2-NBDG) was 0.6401, whereas after unmixing, the *R*^2^ value was 0.9901 (Fig. 1a). Similarly, prior to unmixing, the *R*^2^ value for 2-NBDG (mixed with Bodipy FL C16) was 0.2921, whereas after unmixing, the *R*^2^ value increased to 0.9337 (Fig. 1b). While the spectroscopy phantoms contained two fluorophores, 2-NBDG and Bodipy FL C16, the imaging phantoms contained three fluorophores, 2-NBDG, Bodipy FL C16, and TMRE. The addition of TMRE should not impact spectral unmixing of the other fluorophores, as there is minimal overlap of this reported fluorescence excitation–emission spectra from the other two. The results observed from analyses of widefield fluorescence images were consistent with those observed from phantom spectra Fig. 1c and d. The *R*^2^ values of Bodipy FL C16 and 2-NBDG fluorescence were >0.9 either alone or extracted from mixed phantoms containing two or three fluorophores, and after unmixing, fluorophore intensity measurements match their individual preparation counterparts (Fig. 1). All *R*^2^ values reported for the phantoms are the lines of best fit with the *y*-intercept forced to (0,0).

### A concurrent injection scheme is not significantly different from the gold-standard (multi-day) injection scheme for each fluorophore; optical crosstalk is mitigated by unmixing, and biological crosstalk is mitigated by temporal spacing of TMRE and 2-NBDG

We have established the use of three independent fluorophores for imaging of key metabolic endpoints in vivo: fatty acid uptake via Bodipy FL C16, glucose uptake via 2-NBDG, and mitochondrial membrane potential using TMRE (27–33). The goal of the current experiments was to use the validated excitation–emission pairs established in phantoms for live animal imaging of all three metabolic endpoints in the same session. For all in vivo experiments, fluorophores were diluted in phosphate buffered saline (PBS) and injected retro-orbitally at a combined volume of 100 μL. TMRE was diluted to a 75-μM concentration, 2-NBDG was diluted to a 6-mM concentration, and Bodipy FL C16 was diluted to a 200-μM concentration.

The in vivo imaging studies were performed on normal mammary tissue, and 4T1 tumors orthotopically implanted into mammary tissue. Mice were randomized into experimental groups (*n* = 5 mice for the normal concurrent cohort, *n* = 6 per cohort for all other cohorts) receiving either the concurrent injection scheme (TMRE and Bodipy FL C16 injected in a dual injection with 2-NBDG injected 20 min later) or the gold-standard multi-day injection scheme, where TMRE and 2-NBDG are injected within 20 min of each other (previously validated), and Bodipy FL C16 is injected individually 2 days later to allow for 2-NBDG and TMRE clearance.

**Fig. 2.**
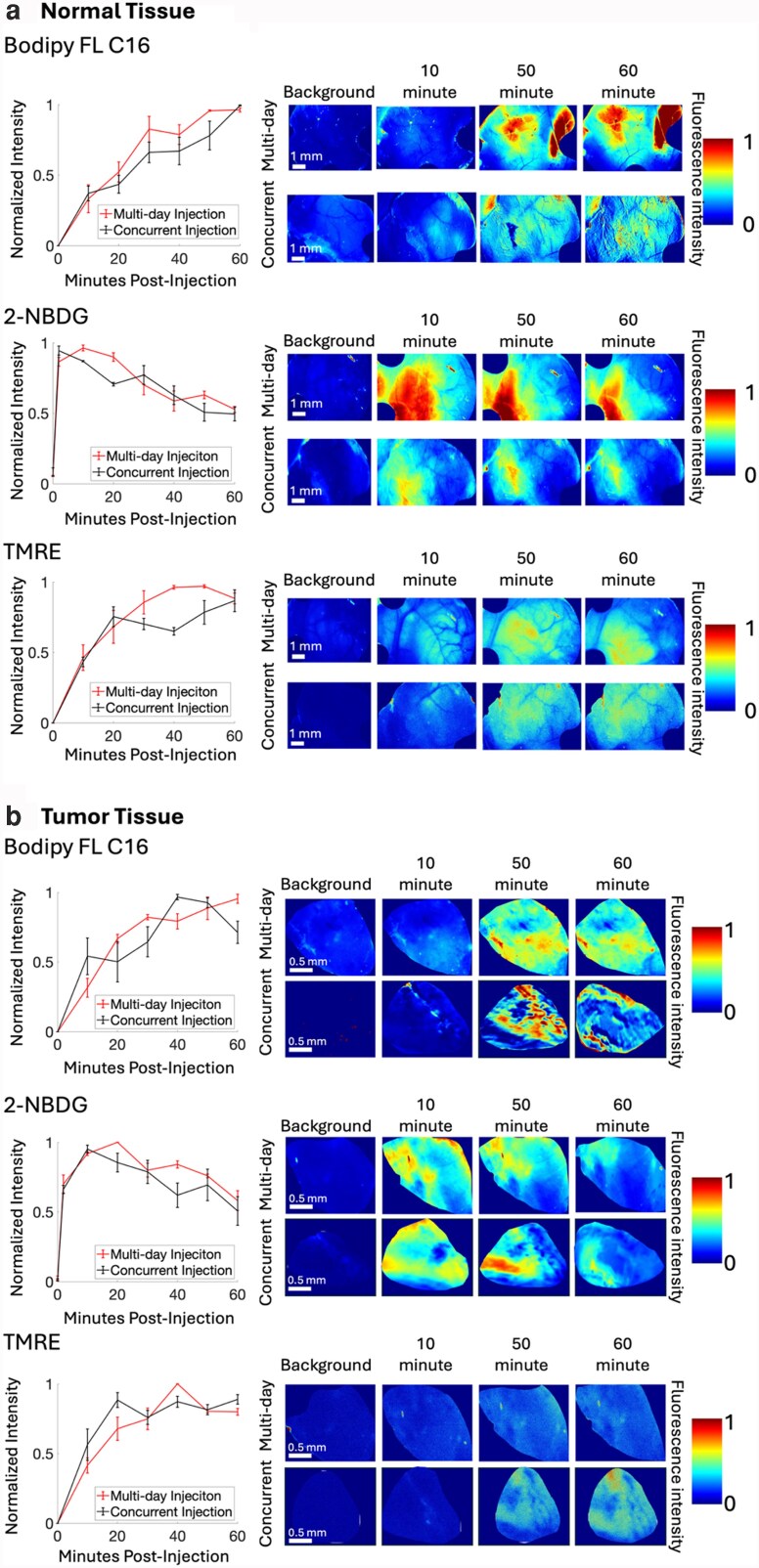
Uptake kinetics and representative images in (a) normal mammary tissue and (b) 4T1 tumor tissue. In both normal tissue (a) and tumor tissue (b), fluorescence intensity of images was obtained every 10 min from 0 to 60 min postinjection of each fluorophore. Multi-day refers to the gold-standard injection scheme in which TMRE (75 μM) is injected 20 min prior to 2-NBDG (6 mM), with both being imaged 60 min postinjection, and Bodipy FL C16 (200 μM) injected individually 2 days later and imaged 60 min postinjection (15). The concurrent injection scheme is defined as Bodipy FL C16 (200 μM) and TMRE (75 μM) injected simultaneously, with 2-NBDG (6 mM) injected 20 min later, and all fluorophores imaged 60 min postinjection. Normalized uptake kinetic curves were constructed by dividing all the time points for each individual animal by the maximum intensity value for that mouse. Each point on the line plots represents the average intensity across *n* = 3 animals. Significance is tested by RM-ANOVA. Error bars are the SEM. Representative images are shown to the right of line plots. Each image is normalized to the maximum value for that image. All images were unmixed prior to analysis.

Uptake kinetics were measured for a subset of mice (*n* = 3) within each experimental group (multi-day and concurrent) for both tumor and normal tissue. Images were obtained every 10 min over a period of 0–60 min postinjection of each fluorophore. The fluorophore uptake kinetic curves were normalized to their maximum value to compare the shape of the curve between the multi-day and concurrent injection scheme. Uptake curves were compared using a repeated measures analysis of variance (RM-ANOVA) test. Figure 2 shows normalized uptake kinetics and representative images in (a) normal mammary tissue and (b) 4T1 tumor tissue. In both tumor and normal tissue, there is a significant difference in fluorophore intensity over time for each fluorophore, showing that fluorophores accumulate in tissue over baseline as expected (*P* < 0.001 for all three fluorophores, tested by RM-ANOVA). For each fluorophore within tumor or normal tissue, there is no significant difference in the kinetic uptake curve over time between the multi-day (gold standard) and concurrent groups (*P* = n.s. for all comparisons made by RM-ANOVA).

**Fig. 3.**
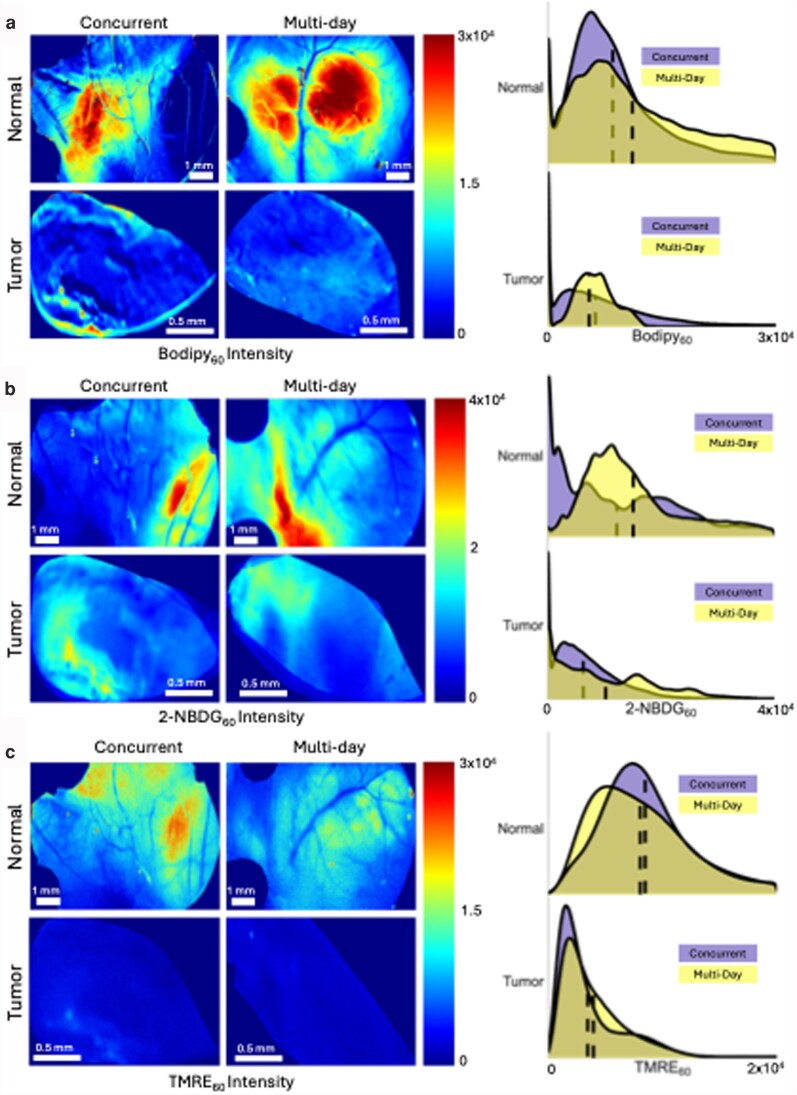
Representative unmixed fluorescence images and PDFs for each fluorophore. a) Bodipy FL C16, b) 2-NBDG, and c) TMRE are shown for the multi-day and concurrent injection scheme for both normal and tumor tissue types. Values are fluorescence intensity in calibrated units (c.u.). Each PDF comprises every pixel in each image across all mice in that group (*n* = 6 mice per cohort for concurrent and multi-day tumor cohorts and multi-day normal cohort, *n* = 5 for the concurrent normal cohort). No significant difference was observed between multi-day and concurrent images across all images in each experimental group, evaluated with a Wilcoxon rank sum test.

Figure 3 shows representative unmixed fluorescence images and probability distribution functions (PDFs) for each fluorophore: (a) Bodipy FL C16, (b) 2-NBDG, and (c) TMRE for the multi-day and concurrent injection scheme for both tumor and normal tissue types. Each PDF comprises every pixel in each image across all mice in that group (*n* = 6 mice per cohort for the concurrent and multi-day tumor cohorts and multi-day normal cohort, *n* = 5 mice per cohort for the concurrent normal cohort). We do not observe a significant difference in pixel intensity between multi-day and concurrent images across all images in each experimental group for each fluorophore when evaluating with a Wilcoxon rank sum test. However, a qualitative evaluation across PDFs shows that normal tissues rely primarily on mitochondrial metabolism, whereas tumors increase glycolytic activity (lower TMRE and Bodipy FL C16) compared with normal tissue.

### Using all three probes concurrently allows us to observe the interplay of metabolic pathways across different tissue types

The goal behind translating our near-simultaneous imaging of all three fluorophores and spectral unmixing is to investigate and utilize the spatial information gained when combining three metabolic endpoints across the same field of view (FOV; 8 mm × 6 mm). Imaging allows us to see heterogeneity in metabolic endpoints across the same tissue region and observe fluorophore clustering by overlaying co-registered images. This allows us to take ratios of pixel intensity, for example TMRE/2-NBDG (the ratio of mitochondrial activity to glucose molecules taken up by a cell) or TMRE/Bodipy FL C16 (the ratio of mitochondrial activity to fatty acid palmitate molecules taken up by a cell). The TMRE/Bodipy FL C16 ratio can be investigated using the concurrent injection scheme, but not the multi-day injection scheme, since Bodipy FL C16 is injected 2 days later after TMRE has already cleared from the animal. Pairwise analysis of fluorophores points towards differences between tumor and normal tissue (Fig. S2); however, this is not sufficient for a complete analysis of metabolic heterogeneity across pathways. Thus, we sought to explore relationships between all three metabolic fluorophores across all animals in each tissue type.

**Fig. 4.**
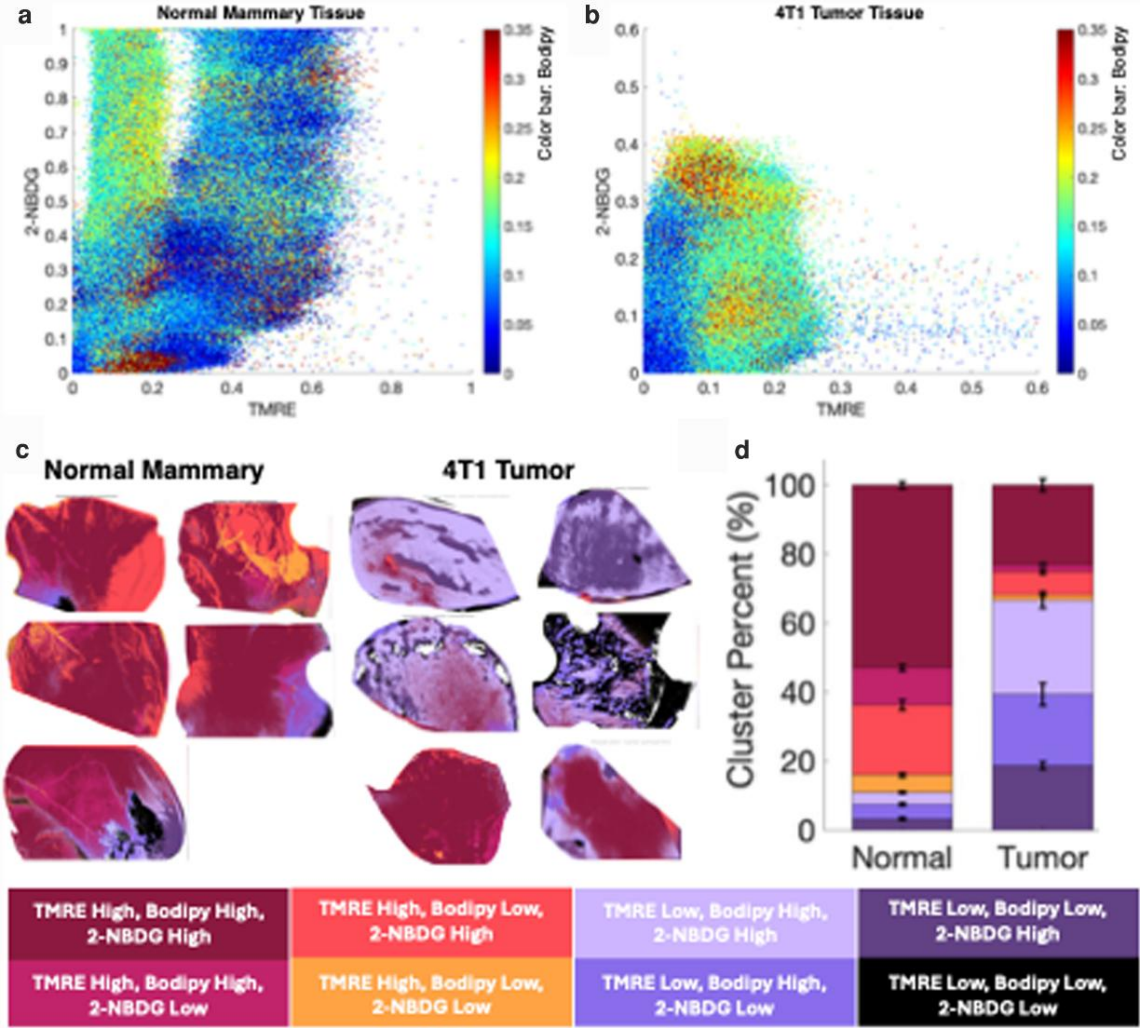
Scatter plots of pixel intensity show the relationship between all three fluorophores (TMRE on the *x*-axis, 2-NBDG on the *y*-axis, and Bodipy FL C16 represented by the color of the points) for normal tissue (a) and 4T1 tumor tissue (b). Scatter plots in (a) and (b) are created using all pixels across all animal images (*n* = 5 animals in the normal cohort, *n* = 6 in the tumor cohort). Analyzing all three metabolic endpoints concurrently allows us to distinguish metabolic signatures between tumor and normal tissue. In (c) and (d), clusters are formed by sorting every pixel in an image into clusters based on its value being either over or under the mean pixel value for each fluorophore. c) Heterogeneity maps for each animal in the study; normal tissue is shown on the left and tumor tissue is shown on the right of (c). Heterogeneity maps are quantified in (d), percent of pixels in each of seven clusters across all animals in the concurrent experimental group (*n* = 5 for normal, *n* = 6 for tumor).

In Fig. 4, we visualize the relationship between mitochondrial activity and two substrates of mitochondrial metabolism by plotting each pixel across all images in the concurrent experimental group. The concurrent injection scheme enables this analysis, as each fluorophore was present in the same tissue at the same time, allowing for co-registration of fluorescent images of our three endpoints. Mitochondrial activity (TMRE) is plotted on the *x*-axis, glucose uptake (2-NBDG) is plotted on the *y*-axis, and fatty acid uptake (Bodipy FL C16) is represented by the color of the points, with warmer colors representing higher Bodipy FL C16 intensity.

Figure 4a shows a scatter plot of all pixels in the normal tissue cohort. We observe an oxidative metabolic phenotype (typical in healthy tissue) where glucose uptake is positively correlated with mitochondrial activity. We see that most points fall along the *x* = *y* diagonal, indicative of a strong positive relationship between mitochondrial activity (TMRE) and glucose uptake (2-NBDG). In addition, examining the color of the points reveals a high Bodipy FL C16 intensity (warmer colors) generally in the low TMRE area of the *x*-axis, where TMRE intensity is <0.2. Observing high Bodipy FL C16 uptake that is not correlated with mitochondrial activity is an expected phenotype in normal tissue, as fats are typically taken up for storage or biosynthesis rather than catabolism in healthy adipose tissue (37). We have observed the same result (elevated Bodipy FL C16 in normal mammary tissue) previously in spectroscopy studies (33).

We next show a scatter plot of all the pixels in the 4T1 tumor tissue group in Fig. 4b. Here, we do not see a strong correlation between 2-NBDG and TMRE. Uptake of glucose (2-NBDG) that is not correlated with an increase in mitochondrial activity suggests a glycolytic phenotype characteristic of tumor tissue. However, we do observe increasingly warmer colors (Bodipy FL C16 intensity) correlating with an increase in the *x*-axis (TMRE, mitochondrial metabolism). This suggests that, in addition to glycolysis, these tumors are using FAO more than normal tissue. Identifying these two metabolic phenotypes in tumor tissue was made possible by having all three fluorophores present in the same tissue at the same time; the FAO metabolic phenotype in tumors would have been missed if we measured only glucose or mitochondrial activity.

Having observed a metabolic shift towards both glycolysis and FAO in tumor tissue compared with normal, we next asked whether we could determine how these metabolic phenotypes present spatially across tumor tissue. To investigate the spatial organization of metabolic signatures and heterogeneity across tissue, we created heterogeneity cluster maps, shown in Fig. 4c. To create the cluster maps, we calculated the mean value of all pixel intensities for each individual fluorophore to create a cutoff value. All pixels that fall above the mean value are labeled as “high” for that fluorophore, while all pixels that fall below that value are labeled as “low” for that fluorophore. The process of labeling all pixels in the dataset as “high” or “low” is repeated for each fluorophore, resulting in eight clusters: four for the case in which TMRE is high: {TMRE_high_, Bodipy FL C16_high_, 2-NBDG_high_}, {TMRE_high_, Bodipy FL C16_high_, 2-NBDG_low_}, {TMRE_high_, Bodipy FL C16_low_, 2-NBDG_high_}, {TMRE_high_, Bodipy FL C16_low_, 2-NBDG _low_}, and four where TMRE is low: {TMRE_low_, Bodipy FL C16_high_, 2-NBDG_high_}, {TMRE_low_, Bodipy FL C16_high_, 2-NBDG_low_}, {TMRE_low_, Bodipy FL C16_low_, 2-NBDG_high_}, {TMRE_low_, Bodipy FL C16_low_, 2-NBDG_low_}. Clusters are represented by colors and displayed in Table 1.

**Table 1.** Eight clusters are created based on whether the intensity of each fluorophore is higher or lower than the average value across that fluorophore across the entire study.

	Bodipy FL C16_high_		Bodipy FL C16_low_	
	2-NBDG_high_	2-NBDG_low_	2-NBDG_high_	2-NBDG_low_
TMRE_high_	TMRE_high_ Bodipy_high_ 2-NBDG_high_	TMRE_high_ Bodipy_high_ 2-NBDG_low_	TMRE_high_ Bodipy_low_ 2-NBDG_high_	TMRE_high_ Bodipy_low_ 2-NBDG_low_
TMRE_low_	TMRE_low_ Bodipy_high_ 2-NBDG_high_	TMRE_low_ Bodipy_high_ 2-NBDG_low_	TMRE_low_ Bodipy_low_ 2-NBDG_high_	TMRE_low_ Bodipy_low_ 2-NBDG_low_

TMRE_low_ clusters are cooler colors and TMRE_high_ clusters are warmer colors.

Looking at the heterogeneity cluster maps, we see concordant information from what we observed in the scatter plots. However, we additionally see the spatial layout of where metabolic phenotypes are occurring within the tissue and can better observe the heterogeneity. We observe that in normal tissue, the dominant phenotype is characterized by high mitochondrial activity and high 2-NBDG levels. Conversely, in tumor tissue, we observe low TMRE uptake, likely due to highly hypoxic tumors resulting from poor vascularization. However, we see areas of high glucose uptake reflecting the expected glycolytic phenotype in tumors. Tumor tissue is more heterogeneous than normal tissue, though both tumor and normal tissue show Bodipy FL C16 uptake.

To quantify these observations, we calculated the percentage of pixels in each cluster, shown in Fig. 4d. {TMRE_low_, 2-NBDG_low_, Bodipy FL C16_low_} clusters are generally areas of low viable cells or bloody areas on tissue (based on reflectance images) and are thus excluded from quantitative analysis. In our stacked bar charts in Fig. 4d, we observe differences in the contribution of different clusters between normal mammary and 4T1 tumor tissue. Approximately 53% of pixels in the normal tissue belong to the same cluster ({TMRE_high_, 2-NBDG_high_, Bodipy FL C16_high_}). The second largest cluster in the normal tissue ({TMRE_high_, 2-NBDG_high_, Bodipy FL C16_low_}) makes up ∼23% of pixels, suggesting that glucose is the predominant substrate of OXPHOS in normal tissue. Taken together, healthy mammary tissue displays a highly oxidative metabolic phenotype (high TMRE indicative of active mitochondria) of two main substrates of OXPHOS in healthy tissue: primarily glucose (for oxidative metabolism) and additionally fats for biosynthesis.

When pooling all the high TMRE and low TMRE clusters regardless of substrate, we see that there is a significant decrease in high TMRE clusters in tumor tissue compared with normal tissue (*P* = 0.0028). Further, the percentage of pixels in the {TMRE_low_, 2-NBDG_high_, Bodipy FL C16_high_} cluster is significantly greater (*P* = 0.0087) in tumor tissue compared with normal tissue. The low TMRE across tumor tissues appears to be indicative of a poorly vascularized tumor environment characteristic of aggressive tumors, triggering a hypoxia-induced glycolytic phenotype. We also observe increased metabolic heterogeneity between and across tumors (Fig. 4c and d). The two largest clusters combined comprise 50% of the data, which includes {TMRE_low_, 2-NBDG_high_, Bodipy FL C16_high_} and {TMRE_low_, 2-NBDG_high_, Bodipy FL C16_low_}, reflecting greater heterogeneity. However, we see intratumor heterogeneity as well as intertumoral heterogeneity; in two of the six tumors, we see most pixels in the high TMRE clusters. Cluster percentages are calculated across the entire group. In general, tumor tissues are predominantly glycolytic, whereas normal tissues are more reliant on mitochondrial metabolism.

## Discussion

This study demonstrates a methodology for concurrently imaging three key metabolic endpoints relevant to preclinical studies of cancer: glucose uptake, fatty acid uptake, and mitochondrial activity. Our results show that fluorophore uptake in the concurrent injection scheme is concordant with what we see in our gold-standard (multi-day) injection. The concurrent injection scheme enables us to provide a more comprehensive report on the relationships between mitochondrial activity and two key drivers of mitochondrial metabolism: sugars and fats. The data show that normal tissue relies on mitochondrial metabolism (37), whereas tumor tissues are predominantly glycolytic. The relationship between substrates is more heterogeneous in tumors compared with normal tissues.

The work reported here builds upon previous publications by our group on optical crosstalk studies of TMRE, 2-NBDG, and Bodipy FL C16 (27–33). Each fluorophore has been robustly validated in vivo to report on a metabolic axis relevant to aggressive breast cancer; Bodipy FL C16 reports on fatty acid palmitate uptake, 2-NBDG reports on glucose uptake, and TMRE reports on mitochondrial membrane potential (27, 28, 31). These baseline studies paved the way for pairwise imaging of a specific substrate (2-NBDG or Bodipy FL C16) and the downstream effect on mitochondrial metabolism (TMRE) (15, 21, 30, 31, 33). We established that there was no inherent optical crosstalk between TMRE and 2-NBDG that needed to be accounted for; however, there was biological crosstalk (29–31). We mitigated biological crosstalk between the two by staggering the administration of 2-NBDG by 20 min after administration of TMRE (29–31). We have demonstrated that combining the two in an in vivo system can reveal whether a tumor primarily relies on glycolysis or glucose oxidation (31). We further established that TMRE and Bodipy FL 16 can be delivered together in the same injection with no observed optical or biological crosstalk that needed to be addressed (33).

Prior to implementing imaging of 2-NBDG and Bodipy FL C16 with TMRE, we first validated our methodologies for spectrally unmixing these overlapping fluorophores using spectroscopy studies (32). The spectroscopy studies allow us to examine the overlap between fluorophores in scattering media and the effectiveness of optical crosstalk correction (32). Furthermore, a subset of wavelengths can be selected to design a system for intravital imaging that faithfully represents the fluorophore contributions determined from the full spectrum. We were able to leverage spectroscopy studies in which the optical crosstalk was addressed for concurrent measurements of all three fluorophores, 2-NBDG, TMRE, and Bodipy FL C16. This study set the foundation for the selection of excitation–emission pairs that would minimize optical crosstalk in the imaging study reported here.

Our results investigating glucose and fatty acid and their contributions to oxidative or nonoxidative metabolism are highly supported by literature (13, 38, 39). High 2-NBDG corresponds to glycolysis when it is inversely related to TMRE and mitochondrial metabolism and when it is positively associated with TMRE. Dysregulated cellular energetics is a hallmark of cancer; in particular, aberrantly high uptake of glucose has been identified as an indicator of cancer (13, 38). When Bodipy FL C16 is positively correlated with TMRE uptake it likely points to FAO. Indeed, literature affirms that reprogrammed cancer metabolism involves an increase in lipid uptake and oxidative metabolism (40, 41). We have independently demonstrated that pre-emptive treatment with etomoxir, a mitochondrial FAO inhibitor that blocks carnitine palmitoyltransferase 1 resulted in a significant decrease in TMRE fluorescence in a Her2 breast cancer model (28). When Bodipy FL C16 is independent of TMRE, it may correspond to fatty acids being taken into the cell for use in biosynthesis (37).

Aside from fatty acid β-oxidation, the main catabolic pathway for long-chain fatty acids, fatty acids such as palmitate can undergo elongation in the cell for use in membrane biosynthesis in both tumor and normal tissue (37). Previous work from our group supports the importance of imaging Bodipy FL C16 as a marker of biosynthesis (33). We have shown elevated TMRE uptake in the normal mammary fat pad of 7- and 8-week-old mice compared with older mice, along with consistently elevated Bodipy FL C16 signal up to 15 weeks of age (33). The mammary fat pad of pubertal female mice (3–8 weeks) is metabolically active, requiring free fatty acids for synthesis as the animal grows (33, 42, 43). This explains our observation of high metabolic activity in the mammary fat pad of our female mice (5–7 weeks of age). Our observation of a glycolytic phenotype in our (untreated) orthotopic 4T1 tumors corroborates these previous findings.

Our cluster maps indicate that tumor tissues are heterogeneous, with regions exhibiting high Bodipy FL C16, 2-NBDG, or both. High Bodipy FL C16 signal may be due to the tumors' proximity to adipocytes. Tumor-surrounding adipocytes, replete in the breast microenvironment, are considered a source of free fatty acids to fuel tumor cell FAO (41, 44). Evidence generated across multiple groups suggests that adipocytes in the vicinity of a tumor decrease in size and lipid content, likely due to lipids being released from adipocytes and transferred to cancer cells (41, 44). One group has shown that the overexpression of an enzyme that breaks down lipids from adipocytes, adipose triglyceride lipase, correlates with tumor aggressiveness, suggesting that the released free fatty acids are then utilized by tumor cells for FAO (40, 41). This suggests that imaging the distribution of adipocytes in addition to Bodipy FL C16 can further confirm this finding.

Heterogeneity maps allow us to make informed inferences. We have discussed extensively the implication that when TMRE is high, this likely points to an oxidative metabolism, and if one substrate or the other is high, that substrate is likely a source of fuel for OXPHOS. When TMRE is high and both substrates are low (1.3% in tumor tissue and 5.1% in normal tissue), this may point to alternative substrates such as amino acids driving oxidative metabolism (45, 46). While fats and sugars provide fuel for mitochondrial metabolism, other metabolic pathways contribute to the breast cancer metabolome. In normal tissue, studies have shown that a maximum of 10% of metabolism is protein fueled under exercise conditions (47). Additional metabolic pathways relevant to aggressive cancer include amino acid metabolism (specifically, glutamine and serine), which our group cannot currently detect (20, 48, 49). Aside from high mitochondrial active phenotypes, a third of the cluster area in our tumor tissues reported low TMRE but high 2-NBDG and high Bodipy FL C16. Fatty acid uptake not linked to FAO has been shown to induce glucose uptake in a study of prostate cancer (50). Having metabolomics to examine this surprising finding would help with interpretation. Therefore, our methodology is complementary to techniques such as metabolomics that can delve deeper into the mechanisms and complex pathways observed using our techniques.

Concurrent imaging of glucose uptake, fatty acid uptake, and mitochondrial metabolism has strong relevance to the imaging of metabolic reprogramming in residual disease. A recent publication by our group used a temporally staggered administration of TMRE and 2-NBDG and reported that a shift from glycolytic to mitochondrial metabolism is a key factor in residual disease and recurrence (15). Due to optical crosstalk between 2-NBDG and Bodipy FL C16, Bodipy FL C16 images were not measured in the same imaging session, limiting the analysis of fatty acid uptake heterogeneity (15). In vivo imaging of a murine model of HER2+ breast cancer with 2-NBDG and TMRE revealed a reliance on non-glucose-driven mitochondrial metabolism in residual disease. The hypothesis that FAO provided an alternative fuel source was tested in the same model using a fatty acid inhibitor treatment, Etomoxir, resulting in prolonged survival (21). Additionally, previous work from our group has shown a glycolytic phenotype in untreated 4T1 tumors and a metabolic shift towards an oxidative phenotype following treatment (21). While these results suggest that FAO is an important fuel source, the inability to combine Bodipy FL C16 with 2-NBDG precluded a direct measurement of fatty acid uptake. Our new methodology enables longitudinal in vivo imaging across the life cycle of a disease model (for example, primary, treated/regressing, residual, and recurrent disease), which can inform time points of interest to further study with techniques such as metabolomics (15).

In conclusion, our technology addresses an important problem—the persistent challenges of how metabolic relationships change in space and time within the same tumor over the tumor life cycle from regression, residual disease, and recurrence. To do so, spatial information and rich datasets of co-registered images with all metabolic endpoints present in the same in vivo system at the same time is necessary. Our method addresses this gap by allowing dynamic, spatially preserved, multi-endpoint metabolic imaging in vivo. This work highlights the importance of not only capturing multiple metabolic endpoints but also investigating their spatial relationships to understand heterogeneity in key substrates and metabolic pathways for energy production in vivo.

## Materials and methods

### Ethics statement

All animal work was carried out in accordance with the recommendations in the Guide for the Care and Use of Laboratory Animals of the National Institutes of Health. All protocols were approved by the Duke University Institutional Animal Care and Use Committee (protocol number A044-24-02). Mice were housed in an on-site housing facility with ad libitum access to food and water with standard light/dark cycles. Mice inoculated with tumors were monitored every other day by palpation and caliper measurement of palpable tumors. Animals were sacrificed at humane endpoints as per ethical guidelines. All experiments were performed under 1–2% isoflurane gas anesthesia, and all efforts were made to minimize suffering.

### Cell culture

All cell lines used herein were 4T1s purchased from the American Type Culture Collection (ATCC) and subjected to mycoplasma testing (ATCC number CRL-2539). 4T1 cells were cultured in RPMI (Gibco, Montgomery County, MD, United States, 11875093) supplemented with 10% fetal bovine serum (Gibco, A3160501) and 1% antibiotics (penicillin/streptomycin; Gibco, 15140122). Cells were incubated at 37 °C with 5% CO_2_ and 95% relative humidity and passaged at ∼80% confluency twice before injection into animals.

### Fluorescent metabolic indicators

TMRE (T669; Thermo Fisher Scientific, Waltham, MA, United States) is a marker of mitochondrial membrane potential (29–31, 34). For in vivo experiments, TMRE was diluted to a 75-μM concentration, a dose optimized prior to achieve a tissue-level TMRE concentration of 50 nM (29–31, 34). This prevents quenching and electron transport chain disruption (29–31, 34). TMRE was used in phantom studies at a concentration of 6 nM to produce measurable and nonsaturated fluorescence for optical crosstalk experiments. TMRE has an excitation peak of 555 nm and an emission peak of 574 nm (29–31, 34).

2-NBDG (N13195; Thermo Fisher Scientific) is a fluorescent analog to glucose which is transported into cells via GLUT inhibitors, like the uptake of natural glucose (27). The final concentration of 2-NBDG for in vivo studies was 6 mM. In phantom studies, 2-NBDG was diluted to a concentration ranging from 2 to 10 μM to produce measurable and nonsaturated fluorescence in a liquid phantom for optical crosstalk investigation. 2-NBDG has an excitation peak of 465 nm and an emission peak of 545 nm (27).

Bodipy FL C16 (D3821; Thermo Fisher Scientific) is a fluorescently labeled palmitate molecule (28). Bodipy FL C16 was diluted to 200 μM for in vivo studies. For liquid phantom studies, Bodipy FL C16 was used at concentrations ranging from 0.2 to 1 μM. Bodipy FL C16 has an excitation peak of 502 nm and an emission peak of 512 nm (28).

Concentrations of TMRE, 2-NBDG, and Bodipy FL C16 are grounded in prior work to ensure the probes are biologically relevant and interpretable (27, 28, 31). Additionally, each probe concentration was optimized to preserve the fidelity of its respective metabolic signal when by itself or with the other probes (30). Longitudinal studies with repeat administration (15) or across multiple imaging sessions (21) show no observable toxicity from the fluorophores at the reported concentrations (15, 21).

### Widefield fluorescence and reflectance imaging

All imaging reported in this manuscript was performed with a widefield fluorescent microscope using LEDs as a light source and an 8.2 MP camera as a detector (16-633; Basler AG, Ahrensburg, Germany) with a variable magnification lens (62-830; Infinity Photo-Optical, CO, United States). The design and geometry of the microscope have been validated and previously reported (51). Light sources used were 470, 450, and 550 nm LEDs (M470D4, M450D4, and M565D2, respectively; Thorlabs Inc., Newton, NJ, United States). Emission filters used were 535, 562, and 592 nm at full width at half maximum (67-019, 67-017, and 67-020, respectively; Edmund Optics, Barrington, NJ, United States). The FOV is 12 mm × 8 mm with a lateral resolution of 10 µm. This system was used for both liquid phantom and in vivo imaging. Imaging software Pylon Viewer (Basler, Ahrensburg, Germany) was used to control image capture.

TMRE was imaged using the 550 nm LED and 586 nm emission filter. The acquisition time was 1 s. To achieve spectral unmixing of Bodipy FL C16 and 2-NBDG using a fluorescent microscopy system, images were captured at four distinct wavelength ranges using a combination of LEDs and emission filters. These were 470 nm LED, 535 nm emission filter; 470 nm LED, 562 nm emission filter; 450 nm LED, 535 nm emission filter; and 450 nm LED, 562 nm emission filter. The acquisition time was 1 s per wavelength pair. Unmixing of Bodipy FL C16 and 2-NBDG signal was performed at postprocessing after the imaging session was completed (described in the Intravital imaging section).

To capture spatial features of both tumor tissue and mammary fat pad, blue light and green light reflectance images were captured at all study endpoints using the same system described above. For blue light reflectance, the 470 nm LED was used with no emission filter. For green light reflectance, the 550 nm LED was used with no emission filter. Reflectance images were captured at 15 ms acquisition time.

Prior to each imaging session, we imaged two standard fluorescent slides to ensure minimal light source variation and calibrate the system to account for day-to-day variation. We used a slide with an excitation of 488 nm and an emission of 519 nm for both 450 and 470 nm LEDs. We used a slide with an excitation of 590 nm and an emission of 650 nm for the 550 nm LED. Light source variation was assessed by imaging the same two fluorescent slides with the same imaging parameters at the start of every imaging session. System calibration consisted of setting the working distance and *x*, *y*, and *z* coordinates of the light source, sample, and detector geometry to ensure a uniform illumination and sharp focus on images of a fluorescent slide based on previously reported methods (51). Tissue autofluorescence was evaluated and determined to be negligible by taking background images at all four wavelength ranges prior to injection of fluorophore. The effect of variable tissue optical properties on the accurate measurement of fluorophores has been investigated and reported previously (32, 33) through the use of variable scattering phantoms, with scattering levels varied across a biologically relevant range, and mixed scattering and absorption phantoms where both scatterer and absorber were varied across a biologically relevant range. In this study, the effect of optical properties was determined by using scattering phantoms and results were consistent with the expected results using the four wavelengths identified from a more extensive set of phantom spectroscopy studies (32, 33).

The working distance and *x*, *y*, and *z* coordinates of the light source, sample, and detector geometry were optimized using previously developed computational methods to ensure uniform illumination across the entire tumor (5 × 5 mm) (51). This is an essential feature that allows for characterization of intratumoral metabolic heterogeneity.

The effect of variable tissue optical properties on the accurate measurement of fluorophores has been investigated and reported previously (32, 33) through the use of variable scattering phantoms, with scattering levels varied across a biologically relevant range, and mixed scattering and absorption phantoms where both scatterer and absorber were varied across a biologically relevant range. In this study, the effect of optical properties was determined by using scattering phantoms, and results were consistent with the expected results using the four wavelengths identified from a more extensive set of phantom spectroscopy studies (32, 33).

### Validation of spectral unmixing of combined 2-NBDG and Bodipy FL C16 phantom images

To enable co-delivery of all fluorophores, we previously developed a spectral unmixing approach that uses a 100-point fluorescence spectrum to separate the contribution of 2-NBDG and Bodipy FL C16 (33) in tissue phantoms and then applied it to quantify these substrates in a 4T1 model of breast cancer. Our phantom studies (Table S1) also demonstrated that varying the optical properties over a wide range had negligible effects on the fluorescent probes (32, 33). To adapt this approach to imaging, we first used the original 100-point spectrum to determine the minimum number of excitation–emission wavelength pairs required to faithfully recapitulate the spectral unmixing achieved with the 100 excitation–emission wavelength pairs in similar tissue phantoms (Table S2).

Guided by the analysis of the fluorescence spectra, we incorporated LEDs and filters into our imaging system that provide four excitation–emission pairs and validated it to spectrally unmix Bodipy FL C16 and 2-NBDG in liquid scattering phantoms (Table S2). The wavelength pairs used were 450 nm LED with a 535-nm emission filter, 450 nm LED with a 562-nm emission filter, 470 nm LED with a 535-nm emission filter, and 470 nm LED with a 562-nm emission filter.

We used the previously developed a linear spectral unmixing strategy on a 100-wavelength fluorescence spectrum to separate the contribution of each of the two overlapping fluorophores, 2-NBDG and Bodipy FL C16 in tissue phantoms (32). To distinguish the signal of Bodipy FL C16 from 2-NBDG when both fluorophores are present, an unmixing strategy was used. The method assumes that the combined spectra of Bodipy FL C16 and 2-NBDG are a linear combination of each individual fluorophore spectrum. We used the phantom spectra to choose a minimum number of excitation–emission wavelength pairs that could faithfully unmix the overlapping spectra of the 2-NBDG and Bodipy FL C16. Finally, we validated the use of the four-point spectrum for linear spectral unmixing in imaging phantoms (Table S2).

First, images at four excitation–emission wavelength pairs were obtained for each individual fluorophore in a scattering phantom to serve as a reference. References are homogenous liquid scattering phantoms made with individual fluorophore preparations, captured with the same imaging parameters and calibration as the mixed or experimental images. The Bodipy FL C16 reference contained 0.05 μM Bodipy FL C16. The 2-NBDG reference contained 3 μM 2-NBDG. Concentrations of fluorophore in reference phantoms were chosen to be biologically relevant and produce outputs within the dynamic range of our system (32). Means across images of homogenous phantoms [scatterer at a fixed reduced scattering coefficient (${\;}_{s}^{\;}$) of 10 cm^−1^ and fluorophore in PBS] were used to construct four-point spectrum for each phantom (system is designed such that intensity is uniform across each image) (35). The scatterer was selected to be representative of that in tissues and was used primarily for the purposes of being able to measure remission from a nontransparent liquid.

Using MATLAB (Mathworks, Natick, MA, United States), a linear least squares fit algorithm was used to generate a coefficient matrix which would minimize the error of Eq. (1):


$$F_{\mathrm{total},\lambda (i,j)}=x_{1}\times F_{\mathrm{Bodipy},\lambda (i,j)}+x_{2}\times F_{2\text{-}\mathrm{NBDG},\lambda (i,j)},$$

where “*F*_Bodipy_” is a reference spectrum for Bodipy FL C16, “*F*_2-NBDG_” is a reference spectrum for 2-NBDG, and *F*_total_ is the fluorescence from a given pixel in the mixed phantom (used to validate the methodology). Variables *x*_1_ and *x*_2_ are the coefficients for Bodipy FL C16 and 2-NBDG, respectively. Coefficients represent the relative contribution to the total signal from each individual fluorophore. They are calculated using lsqcurvefit, a built-in MATLAB function (Mathworks). To convert the algorithm output (coefficient values) to corrected fluorescence intensity values, coefficients were multiplied by the appropriate reference value (either Bodipy FL C16 or 2-NBDG) collected with the 470 nm LED and 535 nm bandpass filter.

### Intravital imaging

For all tumor studies, the 4T1 (ATCC number CRL-2539) murine breast cancer cell line was used to grow orthotopic mammary tumors. Female BALB/c mice (Charles River Laboratories, Raleigh, NC, United States) weighing 25 to 30 g at 5 to 6 weeks of age were used in all animal studies. At 5 weeks of age, each mouse received a 100-μL subcutaneous injection of 30,000 cells in the fourth right mammary fat pad. Tumors were monitored every other day and allowed to grow to a volume of 5 mm × 5 mm before window chamber implantation. Tumor volume was calculated with the formula (length × width^2^)/2, with length recorded as the longest axis and width the shortest. Mammary window chambers were implanted over the fourth right mammary fat pad according to previously established protocol (52). For tumor experiments, the window chamber was implanted such that the tumor was roughly situated in the middle of the window for imaging.

All animal experiments were conducted during the day, and mice were fasted for at least 2 h prior to optical measurements (with ad libitum access to water). Fasting ensured glucose in the body did not compete with fluorophore uptake and signal contrast from the tumor compared with normal tissue (53). To stabilize animals and minimize motion artifacts resulting from respiration, live animals were held in place for the duration of imaging (between 60 and 80 min) using a custom holder to stabilize the window chamber. Animals were anesthetized via isoflurane inhalation (1–2% isoflurane gas mixed with oxygen) throughout the course of the optical measurements. All fluorophores were diluted in PBS (Gibco, 10010023) for injections. A combined volume of 100 μL of the fluorophores was injected intravenously (IV) via the retro-orbital route. The fluorophores circulate in the bloodstream and accumulate in metabolically active tissues. In vivo dosing was optimized previously to achieve tissue-level concentrations that avoid self-quenching (31, 34).

In retro-orbital IV injections the fluorophore circulates in the bloodstream and accumulates in metabolically active tissues, delivered by the circulatory system. Retro-orbital IV injections are more repeatable than tail vein IV injections, as puncturing the tail vein could render it unusable for probe delivery at another time point in the study (54). Finally, the IV route has been used in prior publications by our group for development and validation of TMRE, Bodipy FL C16, and 2-NBDG, and thus is consistent with prior validation studies.

The gold-standard injection strategy is referred to as the multi-day injection and consists of TMRE (75 μM) injected 20 min prior to 2-NBDG (6 mM), with both being imaged 60 min postinjection, and Bodipy FL C16 (200 μM) injected individually 2 days later and imaged 60 min postinjection (15). The concurrent injection scheme is defined as Bodipy FL C16 (200 μM) and TMRE (75 μM) injected simultaneously, with 2-NBDG (6 mM) injected 20 min later, and all fluorophores imaged 60 min postinjection.

At each time point, seven images were collected. Fluorescence images were obtained at the previously mentioned four excitation–emission wavelength pairs. Green light reflectance (550 nm excitation) and blue light reflectance (470 nm) were used to capture the spatial outline of the tumor. The same acquisition time of 1 s was used to capture phantom and in vivo images. Prior to each imaging session, we imaged two standard fluorescent slides to account for day-to-day variations. The slides for the 450 and 470 nm LEDs had an excitation of 488 nm and emission of 519 nm and that for the 550 nm LED had an excitation of 590 nm and an emission of 650 nm. These slides were used to assess light source variation in the wavelength ranges relevant to the fluorophores used. Light source variation was assessed by imaging the two fluorescent slides with the same imaging parameters as those of the preclinical model studies at the start of every imaging session. Though tissue autofluorescence was negligible, background images at all four wavelength ranges were captured prior to injection and subtracted from the fluorescence image of the exogenous fluorophore.

At the start of each study, mice were randomized across cages to minimize batch effects. For experiments comparing the concurrent injection scheme to the gold-standard multi-day injection scheme, a total of 24 mice were randomized into four groups of *n* = 6 per cohort. Experimental groups were (i) normal tissue multi-day injection scheme, (ii) normal tissue concurrent injection scheme, (iii) tumor tissue multi-day injection scheme, and (iv) tumor tissue concurrent injection scheme. The normal tissue concurrent injection scheme cohort was reduced to *n* = 5 for image analysis due to the death of one mouse during imaging. Sixty minutes postinjection time points for Bodipy FL C16, 2-NBDG, and TMRE are referred to as Bodipy_60_, 2-NBDG_60_, and TMRE_60_, respectively. For each of the above experimental groups, a subset of each cohort (*n* = 3 mice per cohort) was also imaged every 10 min from 0 to 60 min to capture fluorophore uptake.

All results are shown as such unless otherwise noted. The spectral unmixing algorithm was advanced pixel by pixel to generate an unmixed result for every pixel in the tissue image (Fig. S1). All Bodipy FL C16 and 2-NBDG data shown are spectrally unmixed via the above methods unless otherwise noted. Absorption and scattering of photons are not corrected from the in vivo images. TMRE has been previously shown to not optically overlap with the excitation or emission of Bodipy FL C16 or 2-NBDG; therefore, accurate measurements of TMRE do not require spectral unmixing. TMRE, 2-NBDG, and Bodipy FL C16 measurements reported here are calibrated intensity values. Intravital images were cropped to remove artifacts such as the sides of the window chamber, as shown in Fig. S2. The tumor tissue borders were identified using the blue light reflectance image and cropped to remove surrounding mammary tissue and window chamber artifacts. All cropping was performed using a MATLAB freehand masking function.

## Supplementary Material

pgag027_Supplementary_Data

## Data Availability

All data may be accessed at the following publicly available online repository: https://doi.org/10.7924/r4z32354z.
