# Supplementary material for: In vivo imaging of metabolic heterogeneity across three endpoints relevant to aggressive breast cancer
Source: PNAS Nexus. 2026 Feb 10;5(3):pgag027. doi: 10.1093/pnasnexus/pgag027 (PMC12964118; doi:10.1093/pnasnexus/pgag027)
Supplement: pgag027_Supplementary_Data [file pgag027_supplementary_data.docx]

Supplementary Section

S1. Scattering Phantoms

Homogenous liquid scattering phantoms were constructed using 1 μm monodisperse polystyrene spheres (1μm diameter, Catalog No. 07310, Polysciences, Warrington, Pennsylvania) (μ_s_′ = 10 cm^−1^) and fluorophore in PBS (Gibco, Montgomery County, Maryland, 10010023). No absorber was added. We constructed sets of phantoms to test our unmixing strategy across varying concentrations of Bodipy FL C16 and 2-NBDG. Recipes for phantoms used for spectroscopy measurements and imaging were constructed according to **Supplementary Table S1** and **Supplementary Table S2**, respectively. Briefly, phantoms contained either Bodipy FL C16 at concentrations 0, 0.2, 0.4, 0.6, 0.8, and 1μM or 2-NBDG at concentrations 0, 2, 4, 6, 8, and 10μM. One set of phantoms was prepared with each fluorophore individually. Additional phantom sets contained the above concentrations with constant concentrations of either Bodipy FL C16 or 2-NBDG or constant concentrations of either Bodipy FL C16 or 2-NBDG and TMRE.

For the validation studies on widefield fluorescence images, scattering phantoms were constructed as outlined in **Supplementary Table S2**. All phantoms contained constant µ_s_’ (10 cm^-1^) and no absorber. Phantoms were either individual preparations of Bodipy FL C16 or 2-NBDG, a mixed preparation of Bodipy FL C16 and 2-NBDG, or a mixed preparation of three fluorophores: Bodipy FL C16, 2-NBDG, and TMRE. While not expected to overlap optically, TMRE (ex: 555 nm, em: 575 nm) was included in optical phantoms as a validation of the final method to combine all three fluorophores concurrently. As shown in **Supplementary Table S2**, Bodipy FL C16 was varied linearly from 0 – 1 µM either individually, mixed with a constant concentration of 2-NBDG (2 µM), or mixed with a constant concentration of 2-NBDG and TMRE (2 µM and 6 nM respectively). 2-NBDG was varied linearly from 0 – 10 µM either individually, with a constant concentration of Bodipy FL C16 (0.8 µM), or with a constant concentration of Bodipy FL C16 and TMRE (0.8 µM and 6 nM respectively).

S2. Pairwise evaluation of fluorophores

**Supplemental Fig. S3 (a)** shows PDFs of the TMRE/2-NBDG ratio and TMRE/Bodipy FL C16 ratio for both tumor and normal tissue (n = 6 animals per cohort for the concurrent and multi-day tumor cohorts and multi-day normal cohort, n = 5 for the concurrent normal cohort) from the concurrent injection scheme. **Supplemental** **Fig. S3 (b)** shows representative heatmap images of the ratio of TMRE/2-NBDG and TMRE/Bodipy FL C16 across the entire FOV of both the tumor and normal mammary tissue in the window chamber. Quantitatively in the PDFs and qualitatively in the representative images, we see differences between tumor and normal tissue. The ratio of TMRE to either 2-NBDG or Bodipy FL C16 for both is lower in tumor tissue than in normal tissue. This is relevant as 2-NBDG and Bodipy FL C16 report on substrates of mitochondrial metabolism (glucose and fatty acid palmitate, respectively). The distribution, represented by the PDF, of both TMRE/Bodipy FL C16 and TMRE/2-NBDG values is statistically significantly different between the tumor tissue and the normal tissue (p < 0.01 for both comparisons) when testing using a two-sample KS test. This result motivated us to investigate the relationship between fluorophores spatially. We clustered all the pixels in each image based on whether they are greater than or less than the global mean for each fluorophore (cutoff value is calculated by pooling all images in the study). All pixels that fall above that value were labeled as “high” for that fluorophore while all pixels that fall under that value were labeled as “low” for that fluorophore. The process of labeling all pixels in the dataset as “high” or “low” was repeated for each fluorophore in a pairwise manner resulting in 4 clusters: TMRE high, substrate high; TMRE high, substrate low; TMRE low, substrate high; and TMRE low, substrate low. This was repeated for both TMRE/2-NBDG and TMRE/Bodipy FL C16. **Supplemental** **Fig. S3 (c and d)** show metabolic heterogeneity through clustering analysis of TMRE/2-NBDG and TMRE/Bodipy FL C16 across tumor and normal tissues. In both TMRE/2-NBDG and TMRE/Bodipy FL C16 cluster maps, significant differences were observed between tumor and normal tissue. We observed a significant difference between tumor and normal in the TMRE high, substrate high cluster (red) for both TMRE/2-NBDG and TMRE/Bodipy FL C16 clustering. While we can observe these quantitatively in **Supplemental** **Fig. S3 (c)**, when we look at images such as shown in **Supplemental Fig. S3 (d)**, we can begin to observe the spatial relationships at play. For example, comparing the TMRE and Bodipy FL C16 clustering to TMRE and 2-NBDG clustering, we see that where one substrate is high, the other is low, while TMRE remains high (as shown with the orange and red clusters in normal tissue). This points towards utilization of one substrate of mitochondrial metabolism. Evaluating our data with a one-sided Wilcoxon Rank Sum test, we observed that there was a significant increase (p = 0.0087) in percent of pixels in the TMRE high, 2-NBDG high cluster for tumor tissue compared to normal tissue. Similarly, we see a significant increase (p = 0.0022) in percent of pixels in the TMRE high, Bodipy FL C16 high cluster for tumor tissue compared to normal tissue. Looking at the TMRE high, substrate low cluster (orange), we observe a significant decrease in tumor compared to normal for TMRE/2-NBDG clustering (p = 0.0087) and TMRE/Bodipy FL C16 clustering (p = 0.0152). There was no significant difference observed between tumor and normal tissue in the TMRE low/substrate high (p = n.s.) cluster for either of the substrates (pink), however, our data shows a trend towards a decrease in percent pixels in TMRE low/substrate high cluster from tumor to normal tissue.

**Supplemental Figure S1.** Optical crosstalk between overlapping excitation and emission spectra of Bodipy FL C16 and 2-NBDG can be mitigated via spectral unmixing. A schematic of our spectral unmixing strategy applied to images. Four images are captured of the same field of view (FOV) in different wavelength ranges: 450 nm LED / 535 nm emission filter; 450 nm LED / 562 nm emission filter; 470 nm LED / 535 nm emission filter; 470 nm LED / 562 nm emission filter. For each set of images, intensity of each pixel is taken from each image and used to create a four point “spectrum” in a pixel-by-pixel loop. This spectrum is used along with reference spectra of individual fluorophores to solve the equation F_total_ = F_Bodipy Reference_ * x_1_ + F_2-NBDG Reference_ * x_2_ where C_1_ and C_2_ represent contributions of each fluorophore to the mixed image.

**Supplemental Figure S2:** Process of cropping to remove artifacts. **(a)** Reflectance image of normal tissue, no cropping has been performed **(b)** Fluorescence image of normal tissue, no cropping has been performed. Red outline shows outline of normal tissue (real signal without artifacts from the window chamber or mouse fur). **(c)** Cropped fluorescence image used for analysis. The window chamber has been removed from the FOV. **(d)** Reflectance image of the entire FOV containing tumor tissue, no cropping has been performed. **(e)** Fluorescence image of entire FOV containing tumor tissue, no cropping has been performed. Red outline shows outline of tumor tissue (real signal without artifacts from the window chamber or surrounding normal tissue). **(f)** Cropped fluorescence image used for analysis. The window chamber and mammary tissue surrounding the tumor has been removed from the FOV.

**Supplemental Figure S3**. **(a)** PDFs of both the TMRE/2-NBDG ratio and TMRE/Bodipy FL C16 ratio (for all animals per cohort) for both tumor and normal tissue from the concurrent injection scheme. **(b)** Representative heatmap images show the ratio of TMRE/2-NBDG and TMRE/Bodipy FL C16 across the entire FOV of both the tumor and normal mammary tissue in the window chamber. **Fig. S3. (c and d)** show metabolic heterogeneity through clustering analysis of TMRE/2-NBDG and TMRE/Bodipy FL C16 across tumor and normal tissues. **(c)** Stacked bars represent the percent of pixels in each of three categories as described above averaged across the tumor and normal groups. **(d)** On the right, representative cluster maps are shown for normal mammary tissue and 4T1 tumor tissue for TMRE/Bodipy FL C16 (top) and TMRE/2-NBDG (bottom).

**Supplemental Table S1:** A series of scattering phantoms were prepared for spectroscopy measurements. Phantoms contained constant µ_s_’, no absorber, and varying concentrations of Bodipy FL C16 and 2-NBDG. Phantoms contained either **(a)** individual preparations of 2-NBDG increasing linearly, **(b)** individual preparations of Bodipy FL C16 increasing linearly, **(c)** dual preparations of 2-NBDG increasing linearly mixed with a constant concentration of Bodipy FL C16, or **(d)** dual preparations of Bodipy FL C16 increasing linearly mixed with a constant concentration of 2-NBDG.

**Supplemental Table S2:** A series of scattering phantoms were prepared for imaging. Phantoms contained constant reduced scattering coefficient (µ_s_’), no absorber, and varying concentrations of Bodipy FL C16, 2-NBDG, and TMRE. Phantoms contained either **(a)** individual preparations of 2-NBDG increasing linearly, **(b)** individual preparations of Bodipy FL C16 increasing linearly, **(c)** dual preparations of 2-NBDG increasing linearly mixed with a constant concentration of Bodipy FL C16, **(d)** dual preparations of Bodipy FL C16 increasing linearly mixed with a constant concentration of 2-NBDG **(e)** preparations of Bodipy FL C16 increasing linearly mixed with constant 2-NBDG and constant TMRE, or **(f)** preparations of 2-NBDG increasing linearly mixed with constant Bodipy FL C16 and constant TMRE.
